# The osteoporotic niche as a metabolic sanctuary for breast cancer dormancy and reactivation: A mechanistic perspective

**DOI:** 10.1016/j.isci.2026.116225

**Published:** 2026-06-03

**Authors:** Haojun Duan, Junpeng Qi, Xianwen Hu

**Affiliations:** 1 Department of Nuclear Medicine, The Affiliated Hospital of Zunyi Medical University Zunyi 563000, People's Republic of China; 2 Second Clinical College, Dalian Medical University, Dalian 116044, People's Republic of China

**Keywords:** Health sciences, Medicine, Medical specialty, Internal medicine, Oncology, Natural sciences, Biological sciences, Systems biology, Cancer systems biology, Cancer

## Abstract

Extended survival in breast cancer marks a major therapeutic success, but the persistence of late-onset bone metastasis remains an unresolved clinical issue. The driver of this latency is not merely the loss of bone mineral density, but rather a fundamental metabolic reprogramming within the aging osteoporotic marrow. Distinct from a simple structural decline, the osteoporotic niche evolves into a “metabolic sanctuary” which is defined as a privileged microenvironmental state that provides disseminated tumor cells with dedicated bioenergetic fuel while simultaneously shielding them from systemic immune and therapeutic pressures. Fueled by marrow adipogenesis, immunosenescence, and disrupted mechanotransduction, this degraded microenvironment actively coaxes dormant tumor cells into reactivation. This mechanistic distinction may offer a plausible hypothesis for the divergent outcomes seen in the D-CARE and ABCSG-18 trials. Pharmacologically preserving bone density alone may prove insufficient if the host’s underlying metabolic dysregulation is left unaddressed. Preventing late recurrence implies a need for a paradigm shift: It requires not simple anti-resorptive approaches, but strategies targeting the metabolic crosstalk that disrupts the bone marrow niche.

## Graphical abstract

**Figure:**
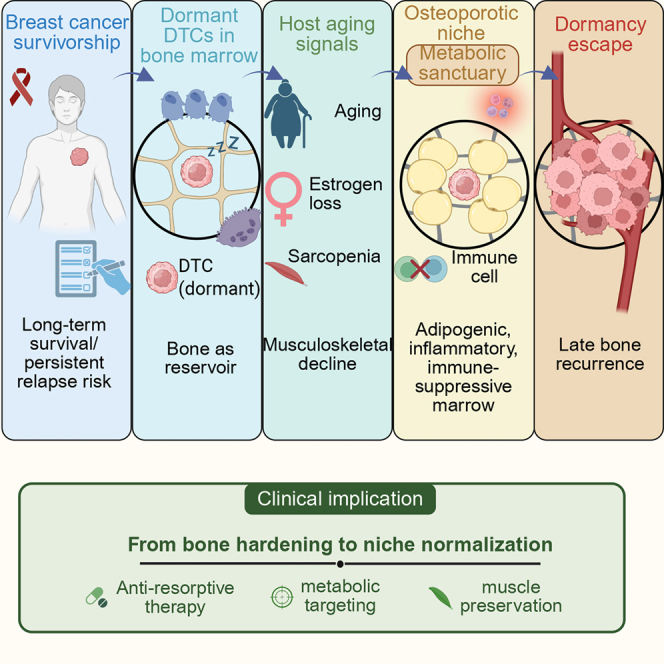

## Introduction

Improvements in screening and adjuvant therapies have increased the 5-year relative survival rate for primary breast cancer to over 90%.^1^ Extended survival inevitably increases the risk for distant relapse.^2^ Among all distant organs, the skeletal system is uniquely susceptible to disseminated tumor cells (DTCs), acting as their primary reservoir. Occurring in approximately 70% of patients with advanced disease, bone metastasis is associated with significant morbidity, skeletal-related events, and reduced overall survival.^3^

The concept of organ-specific metastasis, first framed by Paget in 1889 as the “seed and soil” relationship, remains a cornerstone of cancer biology. C-X-C motif chemokine ligand 12–C-X-C chemokine receptor type 4 signaling directs breast cancer cell migration to the bone marrow, but homing alone cannot account for the long latency typical of late recurrence.^4^ Engineered bone models suggest the marrow is not static but undergoes age-related remodeling. Current evidence demonstrates that metastatic outgrowth does not depend exclusively on intrinsic genetic mutations within the tumor “seed.” Instead, a potential contributor to reactivation is the dynamic, systemic evolution of the microenvironmental “soil” into a metabolically permissive state.^5^ To cohesively integrate these findings, we introduce the concept of the “osteosarcopenic niche.” This pathological musculoskeletal-marrow axis emerges when the synchronized deterioration of skeletal muscle (sarcopenia) and bone mineral density (osteoporosis) jointly creates a metabolic sanctuary for tumor survival. The “metabolic sanctuary” is formally defined as a spatially partitioned compartment within the aged skeleton where the uncoupling of bone remodeling and the expansion of adipocytes create a metabolic landscape tailored to the specific bioenergetic requirements of dormant cancer cells. Beyond mere survival, this sanctuary actively generates localized metabolic byproducts that neutralize extrinsic anti-tumor signals, thereby facilitating immune evasion.^6^ This conceptual framework shifts the paradigm from localized mechanical disruptions to a broader systemic metabolic disorder. It posits that the failure of host-mediated tumor suppression is inextricably linked to the global physiological decline of the aging musculoskeletal system. Recognizing the osteosarcopenic niche as a determinant of late recurrence provides a more comprehensive landscape for understanding how DTCs escape dormancy.^7^ We review recent advances, unresolved challenges, and future perspectives on dormant cancer cell reactivation within the bone marrow niche, toward effective prevention and long-term intervention of breast cancer bone metastasis.

## Clinical validation of the bone-tropic phenotype

Clinical epidemiological and subtype-resolved analyses show that estrogen receptor-positive/hormone receptor-positive breast cancer has a stronger predilection for bone metastasis than triple-negative breast cancer and HER2-enriched disease. Large-scale registry studies further indicate that bone is the dominant metastatic site in HR+/HER2− metastatic breast cancer, whereas triple-negative tumors more frequently relapse in visceral organs such as the lung, liver, and brain.^8^^,^^9^ The estrogen receptor-positive subtype is distinctly defined by prolonged clinical latency and therapeutic resistance. Extensive longitudinal cohort studies demonstrate that even after completing five years of standard adjuvant endocrine therapy, these patients face a persistent and steady risk of late distant recurrence ranging from 13% to 41% during the subsequent fifteen years.^10^^,^^11^ The eventual emergence of minimal residual disease suggests a progressive failure of microenvironmental suppression, an event that temporally coincides with the onset of host osteoporosis and bone marrow senescence.^12^ The metabolic and signaling interactions with the bone niche are likely subtype-dependent.^13^ ER + breast cancer is clinically associated with prolonged latency and late recurrence, and preclinical studies suggest that dormant breast cancer cells can respond to stromal dormancy cues such as TGFβ2 within the bone marrow niche.^14^ In contrast, aggressive triple-negative models more commonly engage osteolytic programs involving tumor-derived factors such as PTHrP and interleukin-8, which stimulate osteoclast activation and bone destruction.^15^ Delineating how the aging marrow transitions from a tumor-suppressive environment to a metabolically permissive sanctuary is critical.

## The osteoporotic niche as a metabolic sanctuary

The establishment of the osteoporotic marrow as a metabolic sanctuary is predicated on two synergistic functional pillars: metabolic privilege and immunometabolic protection. Metabolic privilege refers to the proposed use of adipocyte-derived lipid substrates by DTCs, ensuring their long-term persistence in a nutrient-deprived environment.^16^^,^^17^^,^^18^^,^^19^ Immunometabolic protection involves the localized accumulation of metabolic markers that deactivate effector T-cells, thereby creating a physical-chemical shield against immune-mediated clearance.

In practical terms, osteoporosis is considered here not only as a loss of bone mass but as a niche-level change that supplies metabolic fuel and weakens immune restraint. Although the “seed and soil” hypothesis is widely accepted, establishing the osteoporotic marrow as a true metabolic sanctuary requires demonstrating its temporal and clinical relevance to tumor reactivation.^10^^,^^20^ A nationwide retrospective cohort study of patients with early-stage breast cancer revealed that untreated precancer osteoporosis is significantly associated with an accelerated progression of bone metastasis when it occurs, highlighting the critical role of a degraded bone microenvironment in disease exacerbation.^21^ Preclinical investigations help address the chronological relationship: ovariectomy-induced bone loss occurring prior to tumor introduction can stimulate the proliferation of dormant disseminated breast cancer cells *in vivo*. In these models, estrogen depletion and increased bone turnover promote the growth of dormant cells into overt bone tumors, supporting the interpretation that osteoporotic remodeling may contribute to reactivation rather than merely represent a secondary consequence.^22^ Chronic inflammation and mesenchymal stem cells (MSCs) adipogenic differentiation promote breast cancer cell colonization in the bone marrow. This remodeling further weakens immune surveillance, which leads to the reactivation of dormant tumor cells.

### The adipogenic shift from passive filler to metabolic driver

This niche alteration involves metabolic remodeling driven by a lineage shift in MSCs.^23^ With advancing age and the loss of estrogen, the marrow lineage becomes skewed: Osteogenic differentiation declines while adipogenesis is favored.^12^ Historically considered inert, bone marrow adipose tissue is now recognized as biologically active. Bone marrow adipose tissue functions as a central regulator of metabolic homeostasis, skeletal remodeling, and hematopoiesis, fundamentally influencing the development of bone metastases.^24^ Unlike constitutive marrow fat, osteoporotic bone marrow adipose tissue exhibits a pro-inflammatory phenotype.^25^

Beyond physiological aging, environmental factors also contribute to this phenotypic shift. Endocrine-disrupting chemicals, specifically perfluorohexanesulfonic acid, have been shown to modulate peroxisome proliferator-activated receptor γ signaling, altering MSCs differentiation and promoting adipogenesis.^26^ A similar mechanism is observed in glucocorticoid-induced osteoporosis models, where marrow fat expansion coincides with osteoblast apoptosis.^27^

In the osteoporotic context, bone marrow adipocytes undergo a profound metabolic reprogramming, shifting from inert energy storage to active lipolysis. This adipogenic shift is accompanied by altered lipid-droplet biology and fatty-acid handling in marrow adipocytes. Enzymes involved in lipid-droplet turnover and lipolysis, including adipose triglyceride lipase, hormone-sensitive lipase, and perilipin-associated pathways, regulate fatty-acid mobilization from adipocytes; however, their direction and magnitude in the osteoporotic marrow may vary by model and metabolic context.^28^ The lipidomic profile of the osteoporotic marrow shifts toward pro-inflammatory polyunsaturated fatty acids and specialized signaling lipids, such as lysophosphatidic acid. These lipid species directly engage G-protein coupled receptors on DTCs, providing both the structural building blocks and the energetic fuel required for metastatic survival and subsequent reactivation.^17^ Although adipocyte-to-tumor lipid transfer has been directly demonstrated in ovarian cancer models, comparable direct evidence in breast cancer bone metastasis remains limited.^29^ In the breast cancer bone marrow context, current evidence more cautiously supports a related model: Breast cancer cells can colonize the marrow adipose tissue niche, and adipocyte-derived fatty acids can promote breast cancer cell proliferation and migration in metabolically stressed settings.^18^^,^^19^ Marrow adiposity should be framed as a plausible metabolic contributor to breast cancer bone colonization and reactivation, rather than as a fully proven direct lipid-transfer mechanism in bone metastasis. These findings suggest that marrow adiposity is not merely a marker of skeletal aging, but a metabolically active component that may support metastatic survival.

Emerging bone-metastasis data implicate lipid-handling and ferroptosis-protection programs, including acyl-CoA binding protein (ACBP), in supporting DTC survival within lipid-rich microenvironments. By neutralizing ferroptotic damage during rapid lipid utilization, ACBP ensures the long-term survival and reactivation of disseminated cells within the adipocyte-rich marrow.^30^

### Structural loss of physical restraint and mechanosignaling

The shift from osteogenesis to adipogenesis leads to trabecular bone loss, which increases marrow permeability.^31^ Clinically, T2-corrected Q-Dixon magnetic resonance imaging indicates an inverse correlation between bone mineral density and marrow diffusivity.^32^ Experimental data from microfluidic “bone-on-a-chip” models suggest that this altered architecture increases vascular permeability and impairs normal fluid shear stress, promoting tumor cell extravasation.^33^

Loss of matrix integrity involves more than physical compromise; it disrupts tumor-suppressive signaling. Mechanistically, the structural integrity of a healthy, highly mineralized bone matrix physically constrains DTCs. This rigidity restricts integrin clustering and dampens integrin-mediated focal adhesion signaling, a pathway that can converge on RhoA/ROCK-dependent actomyosin remodeling and YAP/TAZ mechanotransduction.^34^^,^^35^ This lack of cytoskeletal tension securely confines the mechanosensitive transcriptional co-activators, yes-associated protein and transcriptional co-activator with PDZ-binding motif, to an inactive, cytoplasmic state, effectively maintaining proliferative arrest.^34^ Osteoporosis-induced demineralization removes this physical barrier. The resultant structurally compromised and softened osteoid matrix paradoxically alters cellular traction, hyperactivating the RhoA/ROCK tension pathway and unleashing the nuclear translocation of these co-activators. Once localized in the nucleus, the yes-associated protein and transcriptional co-activator with PDZ-binding motif (YAP/TAZ) complex binds to transcriptional enhanced associate domain family transcription factors to transactivate specific pro-metastatic target genes, such as *connective tissue growth factor* and *cysteine-rich angiogenic inducer 61*.^36^ This specific mechanosensory rewiring actively breaks cellular dormancy, switching on pro-proliferative gene programs and driving metastatic recurrence.

### The “inflamm-aging” cascade and immune evasion

Concurrent with these structural changes, the marrow microenvironment exhibits chronic, low-grade inflammation, termed “inflamm-aging”.^37^ The chemokine C-C motif chemokine ligand 3 appears to be a key mediator in this process.^38^ Elevated C-C motif chemokine ligand 3 levels in the senescent niche promote osteoclastic resorption and recruit pro-tumorigenic myeloid cells. This cytokine accumulation suppresses local immune surveillance, facilitating metastatic colonization.

Single-cell analysis of low-bone mineral density niches revealed a pro-tumorigenic microenvironment characterized by extracellular matrix remodeling and immune alteration. Mineral deficiency promoted a shift toward myofibroblastic cancer-associated fibroblasts and increased the density of activated tumor-associated neutrophils and mixed-polarization (nitric oxide synthase 2/arginase 1+) tumor-associated macrophages, contributing to tumor progression.^39^ Senescent osteocytes and adipocytes within the aging niche secrete specific senescence-associated secretory phenotype factors, notably interleukin 6 and various chemokines, which actively subvert local immune surveillance. Rather than causing a generalized inflammatory response, these factors specifically recruit myeloid-derived suppressor cells and polarize tissue-resident macrophages toward an immunosuppressive phenotype.^40^

At the molecular level, this immune shift can be understood as a form of metabolic immune suppression. Osteoporotic niche-derived interleukin 6 engages its cognate receptor on these myeloid infiltrates, strongly phosphorylating the Janus kinase/signal transducer and activator of transcription 3 (JAK/STAT3) signaling cascade. This pathway directly transactivates the expression of arginase-1 and inducible nitric oxide synthase, leading to a profound depletion of local L-arginine and the release of reactive nitrogen species.^41^ This orchestrated immune reprogramming and subsequent amino acid starvation blunt the T cell receptor ζ-chain, driving the upregulation of exhaustion markers such as programmed cell death protein 1. Ultimately, this metabolic starvation leads to the direct exhaustion of cytotoxic CD8-positive T cells, providing robust evidence that the inflamed osteoporotic stroma generates an immunometabolic shield that protects dormant tumor cells from immune clearance.^42^

### Molecular drivers and the loss of tumor suppression

Mechanistic studies indicate that these phenotypic changes result from the dysregulation of specific signaling pathways. This transition involves the increased expression of pro-metastatic factors, such as cytokines and matrix metalloproteinases, alongside a reduction in tumor-suppressive activity. This signaling imbalance drives tumor cells from quiescence to an invasive phenotype. Prior to colonization, breast cancer-derived exosomes containing miR-21 and miR-19a accumulate in the marrow. In conjunction with integrin-binding sialoprotein, these factors condition the niche by promoting osteoclast activity.^43^^,^^44^ Recent studies also implicate the R-spondin 2-leucine-rich repeat-containing G-protein coupled receptor 4 axis in facilitating this enhanced osteoclastogenesis. The resulting local acidification induces transcriptomic changes in dormant cancer cells, leading to their reactivation.^45^^,^^46^

Niche susceptibility involves the loss of protective mechanisms, not merely the accumulation of oncogenic drivers. For instance, the long non-coding RNA Malat1 is essential for maintaining bone integrity. In the context of osteoporosis, the downregulation of this factor increases nuclear factor of activated T cells cytoplasmic 1 transcriptional activity. This leads to increased osteoclast activity and subsequent bone matrix breakdown. The resulting matrix remodeling shifts the bone marrow environment toward a permissive state. By weakening homeostatic barriers, these changes support the recruitment and engraftment of DTCs, promoting bone metastasis.^47^ This process is exacerbated by the age-related decline of Irisin, a myokine that supports osteogenesis mediated by the Wnt signaling pathway.^48^ Emerging bioengineering strategies, such as shock wave-responsive nanocomposites, aim to restore these homeostatic factors.^49^ These findings suggest that effective treatment may require targeting the compromised host microenvironment in addition to the tumor cells.^50^

## Mechanistic subversion of homeostasis

Osteoporotic niche-targeted metastasis is a highly regulated process driven by the subversion of physiological homeostasis. Distinguishing between lifelong physiological bone remodeling and osteoporosis-associated pathological remodeling is essential to understand the exact mechanisms of late recurrence. Under physiological conditions, the continuous, tightly coupled cycle of bone resorption and formation maintains a dynamic microenvironmental equilibrium. This balanced turnover effectively sequesters DTCs in a state of controlled quiescence within the endosteal niche. In the aging or estrogen-depleted skeleton, this remodeling cycle becomes profoundly uncoupled. The pathological acceleration of the osteoclastic resorption phase, without a compensatory increase in osteoblastic formation, does not merely degrade structural integrity; it induces a localized bioenergetic and ionic surge. Rather than being a purely structural process, this uncoupled remodeling may act as a permissive catalyst that weakens physiological suppressive barriers and contributes to a multifaceted intracellular reprogramming cascade associated with tumor dormancy escape.^51^^,^^52^

The assertion that age-related marrow changes drive recurrence is fundamentally rooted in an intricate crosstalk between the tumor cell’s epigenetic landscape and metabolic flux. In the healthy niche, osteoblast-derived suppressive signals maintain high levels of the orphan nuclear receptor NR2F1, an epigenetic gatekeeper that promotes global chromatin repression and sustains a quiescent, non-glycolytic program. The age-related exhaustion of osteoblast lineages in the osteoporotic niche withdraws these critical quiescence-maintaining signals. The loss of these niche signals destabilizes the p38 mitogen-activated protein kinase pathway. The subsequent downregulation of NR2F1 primes the chromatin for transcriptional reactivation, effectively removing the epigenetic brake on tumor proliferation.^53^^,^^54^

**Figure 1:**
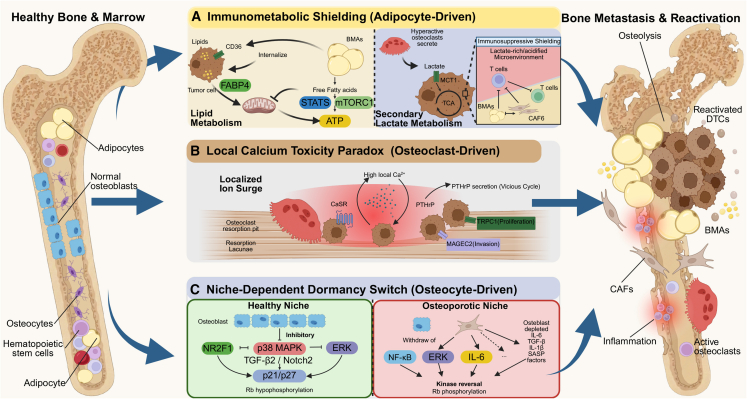
Schematic representation of the mechanisms driving tumor dormancy reactivation in the osteoporotic microenvironment Created in https://BioRender.com. The diagram illustrates the transition from physiological bone remodeling to osteoporosis-related pathological bone remodeling, highlighting three distinct microenvironmental mechanisms that drive this process: (A) Metabolic parasitism and immunometabolic shielding: expanded bone marrow adipocytes (BMAs) act as a direct bioenergetic reservoir, fueling disseminated tumor cells (DTCs) via CD36-mediated lipid internalization and subsequent mitochondrial β-oxidation for ATP production. Concurrently, hyperactive osteoclasts secrete lactate, which DTCs uptake via MCT1 as a secondary carbon source. This acidic environment profoundly inhibits local T cells, while BMAs interact with cancer-associated fibroblasts (CAFs) to further restrict T cell infiltration, forming a protective shield. (B) Local Calcium Paradox: Excessive bone resorption generates extremely high local extracellular calcium concentrations within resorption lacunae. This local calcium surge activates the calcium-sensing receptor (CaSR) on tumor cells, which promotes proliferation via the TRPC1 channel, enhances tumor invasiveness by inducing MAGEC2, and stimulates PTHrP secretion to drive a vicious cycle of osteolysis. (C) Niche-Dependent Dormancy Switch: In a healthy niche, endosteal niche-derived dormancy signals, including Notch2-dependent osteoblastic interactions and MSC-derived TGFβ2, help maintain DTCs in a dormant G0 state. In an osteoporotic niche, the depletion of the osteoblast lineage withdraws these inhibitory signals. Synergizing with accumulated inflammatory factors and senescence-associated secretory phenotype (SASP) factors, this triggers a kinase reversal to an ERK-dominant state. This reversal breaks the cell cycle via Rb phosphorylation and induces metabolic reprogramming, ultimately leading to tumor outbreak.

Crucially, this epigenetic unlocking occurs concomitantly with a massive shift in microenvironmental nutrient availability, facilitating a profound metabolic reprogramming. The pathological expansion of marrow adipocytes and hyperactive osteoclasts bathes the epigenetically primed disseminated cells in a highly reactive milieu. Adipocyte-derived free fatty acids not only fuel mitochondrial beta-oxidation via the upregulation of carnitine palmitoyltransferase 1A, but also actively trigger the signal transducer and activator of transcription 3 and mammalian target of rapamycin complex 1 survival pathways.^16^ The localized accumulation of senescence-associated secretory phenotype factors, particularly interleukin 6 and transforming growth factor β, together with high extracellular calcium released from excessive bone resorption, may shift the intracellular kinase balance. Ultimately, this integrated subversion of homeostasis is manifested through three synergistic functional pillars that collectively define the metabolic sanctuary: the evolution of metabolic parasitism into immunometabolic shielding, the exploitation of the localized calcium signaling paradox, and the niche-targeted orchestration of the dormancy-to-reactivation switch.^55^ As comprehensively mapped in Figure 1, this triple-mechanism cascade visually captures how the deterioration of the osteoporotic microenvironment structurally and metabolically orchestrates the transition from tumor dormancy to overt metastasis.

### Evolution of metabolic parasitism into immunometabolic shielding

Rather than drawing parallels from non-osteotropic malignancies, recent lineage tracing and co-culture models specific to breast cancer have demonstrated that bone marrow adipocytes are metabolically active drivers of osteotropism. In the osteoporotic marrow, these adipocytes may act as metabolic substrate providers for breast cancer DTCs.^17^^,^^18^^,^^19^ Specifically, breast cancer cells upregulate the fatty acid transporter CD36 to internalize adipocyte-derived lipids. To avert lipotoxicity, these cells concurrently upregulate specific fatty acid binding proteins, such as fatty acid binding protein 4, facilitating the rapid shuttling of these lipids into the mitochondria for beta-oxidation. This metabolic coupling may provide energetic support that helps breast cancer cells survive in the nutrient-deprived osteoporotic microenvironment, although direct evidence for this process in breast cancer bone metastasis remains incomplete.^16^ Beyond lipid transfer, the osteoporotic niche fosters a complex metabolic symbiosis between bone-resorbing cells and tumor cells. Hyperactive osteoclasts, a pathological hallmark of osteoporosis, exhibit heavily upregulated aerobic glycolysis to meet the extreme energy demands of bone matrix degradation. This metabolic shift results in the massive secretion of extracellular lactate. DTCs can import this localized lactate shuttle via monocarboxylate transporter 1, utilizing it as a secondary carbon source for the tricarboxylic acid cycle to endure nutrient-deprived dormancy. Concurrently, this lactate-rich and acidified microenvironment profoundly suppresses local T cell proliferation and effector functions, solidifying an immunometabolic shield that protects dormant cells from immune clearance.^56^

Conversely, exogenous chemerin supplementation effectively inhibits breast cancer cell invasion.^57^ Studies using pharmacologic agents, including AN698 and peroxisome proliferator-activated receptor γ antagonists, indicate that this imbalance is driven by the tumor microenvironment.^58^ BMAs interact with cancer-associated fibroblasts to restrict T cell infiltration, creating an immunosuppressive environment.^59^ While the concept of an immunosuppressive environment is biologically compelling, validating the specific interactions among bone marrow adipocytes, cancer-associated fibroblasts, and the immune system in breast cancer remains methodologically challenging due to the inherent limitations of standard *in vivo* models.

Traditional xenograft models, such as the injection of human MDA-MB-231 cells into non-obese diabetic/severe combined immunodeficient mice, fundamentally lack a functional adaptive immune system, rendering them unsuitable for evaluating T cell exclusion. Conversely, immunocompetent syngeneic models, including the introduction of murine 4T1 cells into BALB/c mice, possess intact immunity but typically utilize young animals. These younger hosts fail to faithfully recapitulate the adipocyte-rich, osteoporotic bone marrow characteristic of elderly human patients.^60^ The specific methodologies used to induce osteoporosis and metastasis impose inherent interpretative constraints. For instance, while ovariectomy is the gold standard for acute postmenopausal modeling, it primarily simulates rapid estrogen withdrawal rather than the chronic, cumulative “inflamm-aging” and progressive mechanical deterioration seen in geriatric osteoporosis. Similarly, standard bone metastasis models utilizing intracardiac or intratibial inoculation, while effective for studying overt colonization, bypass the early systemic steps of the metastatic cascade. The resulting artificially high tumor burden within the marrow may override the subtle niche-derived suppressive signals, potentially masking the intricate mechanisms that govern true clinical latency.^60^

To navigate these experimental bottlenecks, researchers are increasingly shifting toward more physiologically relevant platforms. One promising approach involves utilizing ovariectomized immunocompetent mice, which successfully bridge the gap between hormonal bone loss and active immune surveillance.^61^ The implementation of tissue-engineered humanized bone-in-mice models provides human bone scaffolds that serve as a species-specific metabolic sanctuary for DTCs.^62^ Ultimately, integrating these approaches with humanized immune-reconstituted mice allows for the observation of immunometabolic shielding in a context that closely mirrors clinical pathophysiology. Employing these advanced models will be important for testing whether pharmacological targeting of bone marrow adipocytes may re-sensitize the osteoporotic niche to immune clearance.^63^

It is important to acknowledge the physiological distinctions between rodent and human bone marrow dynamics. While red-to-yellow marrow conversion in humans is a gradual, centripetal process driven by aging, rodent models often rely on acute induction. The fundamental metabolic signaling pathways-the interplay between adipocytes and tumor cells via FABP4-appear phylogenetically conserved, suggesting that mechanistic insights from these models remain relevant to human pathophysiology.

### The calcium paradox of systemic deficiency and local toxicity

Epidemiological meta-analyses indicate that dietary calcium and Vitamin D deficiency correlate with increased malignancy risk.^64^^,^^65^^,^^66^ This creates an apparent paradox, as large-scale randomized trials, notably Women’s Health Initiative, failed to demonstrate a significant reduction in breast cancer incidence with systemic supplementation.^67^ This discrepancy highlights the critical independence of the local ionic microenvironment from systemic homeostasis. Even among patients with normal serum calcium levels, bone resorption associated with osteoporosis markedly elevates extracellular calcium concentrations precisely within the resorption lacunae-the exact microenvironment where dormant tumor cells are localized. Systemic supplementation maintains serum levels but likely fails to correct the steep, local ionic gradients generated by osteoclasts.^68^

It is this local calcium elevation, rather than circulating levels, that acts as a potent driver of metastasis. High local calcium concentrations activate the calcium-sensing receptor on tumor cells.^69^ This activation induces a phenotypic shift via multiple downstream effectors: it promotes proliferation and stimulates the secretion of PTHrP, thereby driving the “vicious cycle” of osteolysis.^70^^,^^71^^,^^72^ Calcium-sensing receptor signaling via transient receptor potential canonical 1 (TRPC1) channels has been shown to facilitate proliferation.^73^ Consistent with the “coupling theory”, this local hypercalcemia also induces melanoma-associated antigen C2 (MAGEC2) expression, which potentiates invasion without causing toxicity.^74^ The tumor co-opts host compensatory mechanisms, where parathyroid hormone-mediated resorption aims to maintain serum homeostasis but fuels local expansion through high-calcium microdomains.

### Niche-dependent control of the dormancy switch

Breast cancer often features a long clinical latency and a risk of late recurrence.^75^ This phase is considered a state of cellular dormancy regulated by the microenvironment, rather than simple metabolic inactivity. In the bone marrow niche, DTCs execute cell-cycle arrest through defined signaling cascades to maintain homeostasis with the surrounding microenvironment. These dormant cells can persist at distant sites for years after the primary tumor is removed. Subsequent reactivation, often triggered by external factors, eventually leads to metastatic disease.^76^

Breast cancer DTCs can exploit hematopoietic stem cell-like endosteal niches to enter or maintain dormancy. In breast cancer models, Notch2HIGH cells show features of hematopoietic stem cell mimicry and preferential interaction with the endosteal niche, supporting a direct role for Notch2 signaling in bone dormancy and mobilization.^77^ Separately, breast cancer DTC dormancy has been linked to bone marrow NG2+/nestin+ mesenchymal stromal cells and TGFβ2-dependent signaling, which supports a quiescent state through dormancy-associated kinase programs.^14^ The specific Wnt5a-ROR2 axis should be interpreted more cautiously in the breast cancer setting. While Wnt5a/ROR2-mediated dormancy has been demonstrated most directly in prostate cancer bone models, direct evidence that Wnt5a maintains breast cancer dormancy in bone remains limited. Therefore, in this review, Wnt5a is discussed as a potentially analogous osteoblastic niche signal rather than as an established breast cancer bone-metastasis mechanism. This microenvironmental suppression effectively downregulates aerobic glycolysis and enforces a state of severe metabolic austerity, strictly maintaining the cells in a quiescent G0 arrest.^78^

A key question concerns the mechanism of reactivation. Emerging data suggest that niche senescence is a primary driver, rather than intrinsic tumor evolution. In the healthy state, osteocytes maintain quiescence via Connexin-43 gap junctions.^79^^,^^80^ In mechanically compromised conditions such as osteoporosis or obesity, this regulation is impaired.^61^ This contrasts with the physiological response in younger bone, where mechanical loading supports the suppressive barrier.^25^ Additionally, the aging matrix accumulates inflammatory factors that may override Wnt5a-mediated dormancy signals.^81^ Supported by three-dimensional co-culture and imaging studies, these findings necessitate an evolution of the “Niche Failurehypothesis”.^82^^,^^83^

Therefore, dormancy escape represents a dynamic co-evolution: The failing osteoporotic niche acts as the external catalyst that actively drives profound intrinsic epigenetic plasticity within the DTCs. As the aging matrix loses mechanical integrity and accumulates inflammatory factors such as interleukin-1 beta, the resulting nuclear factor kappa B and ERK hyperactivation disrupts this dormant kinase balance, shifting it toward ERK dominance (p38^LOW^/ERK^HIGH^).^84^ This pathway reversal induces cyclin-dependent kinases 4 and 6 -mediated Rb phosphorylation, propelling the cell past the G1/S checkpoint. The previously enforced metabolic austerity is broken. This shift is accompanied by epigenetic plasticity and metabolic reprogramming, rather than by a single linear pathway. Evidence from dormancy models indicates that escape from quiescence can involve changes in chromatin state and increased expression of metabolic programs, including glycolytic or lipid-handling pathways, but the direct coupling of these specific events in breast cancer bone metastasis remains incompletely defined.^16^^,^^54^ Ultimately, recurrence results from the synergistic interplay between microenvironmental deterioration and the tumor’s acquired metabolic aggression.^16^ To systematically translate these mechanistic insights into clinical utility, Table 1 delineates the key molecular drivers within the osteoporotic niche alongside their corresponding experimental and clinical therapeutic targets.

**Table 1:** Key molecular drivers of the osteoporotic niche and potential therapeutic targets

Functional category	Molecular target	Mechanism of action in the osteoporotic niche	Therapeutic implication/clinical relevance	Reference
Lipid metabolism	CD36	a fatty acid transporter that mediates the uptake of exogenous lipids released by marrow adipocytes, fueling tumor growth via metabolic adaptation.	potential target for blocking lipid acquisition; high expression correlates with metastasis.	Wang et al.^13^
	FABP4	transports intracellular lipids to mitochondria for β-oxidation, preventing lipotoxicity while providing energy for colonization.	pharmacologic inhibition may starve tumor cells by disrupting the metabolic coupling between adipocytes and tumor cells.	Wang et al.^13^
	PPARγ	driven by aging and PFHxS, its activation skews MSC differentiation toward adipogenesis at the expense of osteogenesis.	PPARγ antagonists may prevent the expansion of the adipogenic niche.	Fidler,^20^ Yeh and Ramaswamy^53^
	Leptin/Visfatin	pro-tumorigenic adipokines that promote tumor invasion via STAT3-mediated cytoskeletal reorganization.	highlights the active signaling role of marrow fat beyond passive storage.	Aragon et al.,^85^ Chan et al.^86^
	CPT1A	upregulated by adipocyte-derived free fatty acids, it acts as a rate-limiting enzyme fueling mitochondrial beta-oxidation to support survival.	potential metabolic target to disrupt lipid utilization in the nutrient-deprived niche.	Wang et al.^13^
	STAT3 & mTORC1	crucial survival pathways actively triggered by the influx of adipocyte-derived free fatty acids.	key intracellular signaling hubs maintaining tumor cell survival and driving reactivation.	Wang et al.^13^
Lactate metabolism & Shielding	MCT1	imports localized extracellular lactate secreted by hyperactive osteoclasts, utilizing it as a secondary carbon source for the TCA cycle.	target to disrupt the metabolic symbiosis between tumor cells and osteoclasts.	Lawson et al.^51^
	Chemerin	an adipokine whose exogenous supplementation effectively inhibits breast cancer cell invasion.	potential tumor-suppressive microenvironmental modulator.	Croucher et al.^52^
Calcium signaling	CaSR	senses high extracellular Ca^2+^ concentrations within resorption lacunae; activation drives proliferation and PTHrP secretion.	explains why systemic calcium levels do not reflect local microenvironmental toxicity; targets the “vicious cycle”.	Chen et al.,^64^ Lipkin and Newmark,^65^ Peterlik et al.,^66^ Chlebowski et al.^67^
	TRPC1	a cation channel downstream of CaSR signaling; facilitates calcium entry to drive tumor cell proliferation.	a specific downstream effector of the calcium paradox mechanism.	Peterlik and Cross^68^
	MAGEC2	induced by local hypercalcemia; potentiates tumor invasion without causing cellular toxicity.	links the high-calcium microenvironment to the acquisition of an invasive phenotype.	Saidak et al.^69^
Dormancy maintenance	Connexin-43	forms gap junctions with tumor cells to maintain quiescence. This physical restraint is lost in the osteoporotic/mechanically compromised niche.	preservation of osteocyte network integrity is crucial for maintaining dormancy.	Gomis and Gawrzak,^75^ Duong et al.^76^
	Wnt5a	a non-canonical Wnt ligand that maintains tumor cells in a non-proliferative, dormant state. Its signal is often overridden by inflammation.	suggests that “normalizing” the niche requires restoring osteoblastic signaling.	El Hiani et al.,^73^ Capulli et al.^77^
	Irisin	a myokine that supports Wnt-mediated osteogenesis. Its age-related decline (Sarcopenia) weakens the bone niche.	links sarcopenia to bone metastasis; suggests exercise or Irisin mimetics as potential adjuvant strategies.	Yuan et al.^43^
	Malat1	maintains bone integrity; its downregulation in osteoporosis increases NFATc1 activity, leading to matrix breakdown.	an upstream regulator of bone stability; loss triggers a permissive state.	Wu et al.^42^
	NR2F1	an epigenetic gatekeeper that promotes global chromatin repression and sustains a quiescent, non-glycolytic program; downregulated upon osteoblast exhaustion.	crucial epigenetic target; preserving its expression maintains the epigenetic brake on proliferation.	Xing et al.,^48^ Li et al.^49^
	ROR2	non-canonical receptor on tumor cells that engages osteoblast-derived Wnt5a to sustain a dormancy-enforcing kinase balance.	key receptor for niche-dependent dormancy; loss of signaling permits reactivation.	El Hiani et al.^73^
	p38 MAPK/ERK ratio	intracellular kinase balance, where p38HIGH/ERKLOW maintains dormancy; microenvironmental deterioration flips this to ERK dominance.	core biochemical switch reflecting the transition from a suppressive to a permissive microenvironment.	El Hiani et al.,^73^ Chen et al.^80^
	p21, p27, Rb & CDK4/6	cell cycle inhibitors (p21/p27) lock Rb in a hypophosphorylated state (G0 arrest); niche degradation induces CDK4/6-mediated Rb phosphorylation.	execution mechanism of cell cycle reactivation; targets for preventing the G1/S transition.	El Hiani et al.,^73^ Chen et al.^80^
Microenvironment remodeling	CCL3	recruit pro-tumorigenic myeloid cells and promotes osteoclastic resorption, suppressing local immune surveillance	A key mediator of immune evasion in the aged marrow.	(34)
	Notch2	upregulated to mimic HSC, allowing tumor cells to occupy the endosteal niche.	Describes the specific mechanism for initial engraftment and survival.	Johnson et al.^72^
	RANKL	essential mediator of osteoclastogenesis; upregulated in the pro-inflammatory niche to drive bone resorption.	Target of Denosumab; efficacy depends on dosing schedule.	Monteran et al.,^40^ Coleman et al.^87^
	IL-6 & TGF-β (SASP factors)	factors) Senescence-associated secretory phenotype factors that synergize with high calcium to tip the intracellular kinase balance and subvert local immune surveillance.	Key drivers of inflamm-aging and therapeutic targets for niche normalization.	Panciera et al.,^35^ Hofbauer et al.^50^
	IL-1β & NF-κB	inflammatory factors accumulate in the aging matrix that hyperactivate NF-κB and disrupt the dormant kinase balance.	inflammatory drivers of tumor reactivation and potential targets for maintaining dormancy.	Chen et al.^80^

Abbreviations: FABP, fatty acid-binding protein; PPARγ, peroxisome proliferator-activated receptor gamma; MSC, mesenchymal stem cell; CPT1A, carnitine palmitoyltransferase 1A; ACBP, acyl-CoA binding protein; STAT3, signal transducer and activator of transcription 3; mTORC1, mammalian target of rapamycin complex 1; MCT1, monocarboxylate transporter 1; TCA, tricarboxylic acid; CaSR, calcium-sensing receptor; PTHrP, parathyroid hormone-related protein; TRPC1, transient receptor potential canonical 1; MAGEC2, melanoma-associated antigen C2; YAP, Yes-associated protein; TAZ, transcriptional co-activator with PDZ-binding motif; RhoA, Ras homolog family member A; ROCK, Rho-associated protein kinase; NFATc1, nuclear factor of activated T cells 1; NR2F1, nuclear receptor subfamily 2 group F member 1; ROR2, receptor tyrosine kinase-like orphan receptor 2; MAPK, mitogen-activated protein kinase; ERK, extracellular signal-regulated kinase; Rb, retinoblastoma; CDK, cyclin-dependent kinase; CCL3, C-C motif chemokine ligand 3; HSC, hematopoietic stem cell; RANKL, receptor activator of nuclear factor kappa-B ligand; IL, interleukin; TGF-β, transforming growth factor beta; SASP, senescence-associated secretory phenotype; NF-κB, nuclear factor kappa B.

## Clinical evolution from bone protection to microenvironmental modulation

There is a discrepancy between the clear mechanisms identified in preclinical models and the outcomes observed in clinical practice. This translation gap is highlighted by conflicting trial data and the varying influence of metabolic factors. Preventing metastasis may require strategies beyond simple osteoclast blockade. Effective management likely involves addressing the host’s systemic hormonal and metabolic status.

### Validating the seed and soil interaction via the estrogen context

Early clinical trials evaluating bisphosphonates produced inconsistent results regarding the “seed and soil” hypothesis. A clinically important link between host endocrinology and therapeutic efficacy was supported by the Early Breast Cancer Trialists’ Collaborative Group. Antiresorptive agents conferred a survival advantage exclusively within a low-estrogen environment, underscoring the hormone-dependent nature of the metastatic niche.^88^ These data indicate that estrogen levels regulate the bone marrow environment. It also suggests that the lowest estrogen state caused by certain treatments is likely a requirement for bone-targeted drugs to be effective. When estrogen levels are low, the bone marrow undergoes specific biochemical and structural changes. These changes appear to make drug targets more accessible and help activate anti-tumor and bone-protective responses. Beyond supporting the “seed and soil” hypothesis, these findings suggest that assessing estrogen status is vital for timing bone-targeted therapy and improving breast cancer outcomes.

Longitudinal follow-up from the adjuvant zoledronic acid to reduce recurrence (AZURE) cohort reinforced this, distinguishing a “therapeutic window” driven not by chronological age, but by the reproductive senescence of the bone marrow.^89^ Similarly, the Austrian Breast and Colorectal Cancer Study Group (ABCSG) −12 trial enrolled 1,803 patients with early breast cancer who received chemical ovarian suppression with goserelin, with or without additional zoledronic acid. Compared with the AZURE trial, these findings indicate that in premenopausal settings, chemical ovarian suppression is a prerequisite for unlocking the anti-metastatic potential of zoledronic acid.^90^ In conclusion, these results support the role of the bone marrow microenvironment in skeletal protection and adjuvant therapy. Bone remodeling during low-estrogen states influences the efficacy of adjuvant bone-targeted agents. Within this low-estrogen environment, altered bone turnover may create a “therapeutic window” for drugs such as bisphosphonates or denosumab, leading to more effective osteoclast inhibition and a reduction in metastatic progression. Evaluating menopausal and endocrine profiles is vital to optimizing bone-targeted therapy and outcomes in patients with breast cancer.

Regarding the choice of agent, the SWOG Cancer Research Network S0307 trial compared three distinct bisphosphonates: zoledronic acid, clodronate, and ibandronate. The study found no significant difference in disease-free survival among the treatment arms, suggesting a “class effect” driven by osteoclast inhibition rather than the pharmacokinetics of specific molecules.^91^ In light of these findings, the American Society of Clinical Oncology (ASCO) and European Society for Medical Oncology (ESMO) clinical practice guidelines establish adjuvant bisphosphonates as standard of care for postmenopausal patients and those receiving ovarian function suppression.^92^^,^^93^^,^^94^ In contrast, attempts to identify predictive tumor biomarkers, specifically MAF amplification, have failed to demonstrate clinical utility.^95^ This indicates that host menopausal status remains a more reliable predictor of therapeutic benefit than tumor-intrinsic genetic markers.

### The divergence of biological blockade and clinical efficacy in RANKL inhibition

Following the success of bisphosphonates, the receptor activator of nuclear factor kappa-B ligand (RANKL) inhibitor denosumab was investigated for its potential to prevent metastasis. The conflicting trajectories of the D-CARE and ABCSG-18 trials are frequently discussed in this context. Direct comparisons must be interpreted with caution, as these two trials differed substantially in their patient populations, baseline endocrine contexts, specific primary endpoints, and overall dosing regimens. Consequently, attributing their divergent outcomes exclusively to bone-niche biology would be overly deterministic. While these clinical variables independently influence patient survival, the “niche normalization versus bone hardening” concept provides a plausible adjunctive biological hypothesis. From this perspective, the lack of metastasis prevention in the intensive dosing schedule of D-CARE suggests that aggressive pharmacological “hardening” of the bone, despite increasing BMD, may not equate to niche normalization.^87^^,^^96^

Conversely, the success of the physiologic dosing regimen in ABCSG-18 implies that preserving homeostatic remodeling, rather than completely obliterating it, might be more conducive to maintaining tumor dormancy.^97^ Long-term follow-up substantiated the durability of this benefit, demonstrating superior bone-protective effects and fewer adverse events, with a substantially reduced incidence of osteonecrosis of the jaw relative to high-dose protocols.^98^^,^^99^ The biological rationale connecting a homeostatic bone niche to the maintenance of tumor dormancy is increasingly substantiated by clinical translational immunology studies. High-intensity receptor activator of nuclear factor kappa-B ligand blockade, as utilized in the D-CARE protocol, induces a profound depletion of both osteoclasts and their coupled osteoblasts. Because mature osteoblasts are an important physiological source of dormancy-enforcing signals, completely freezing bone turnover paradoxically eliminates this critical suppressive barrier. Clinical translational analyses indicate that aggressive, non-physiological receptor activator of nuclear factor kappa-B ligand inhibition inadvertently impairs local T-cell-mediated anti-tumor immunity, creating an immunometabolic void that facilitates metastatic outgrowth.^100^ Conversely, physiologic dosing regimens, such as those in the ABCSG-18 trial, preserve a basal level of osteoclast-osteoblast coupling, continuously replenishing the functional osteoblastic niche required to enforce long-term tumor quiescence without compromising immune surveillance. To further elucidate this divergence in clinical efficacy, Table 2 provides a comparative analysis of these major adjuvant breast cancer trials, highlighting how distinct dosing regimens and patient baseline characteristics may influence therapeutic outcomes.

**Table 2:** Comparison of dosing strategies, clinical phenotypes, and mechanistic implications across major adjuvant breast cancer trials

Trial name	Dosing strategy	Clinical phenotype & Niche proxy		Mechanistic implication: the “Sanctuary” hypothesis	Reference
		Outcome	Niche proxy		
D-CARE/AZURE	high-intensity blockade: frequent, high-dose administration (e.g., Q4W) aimed at maximum suppression.	no survival benefit observed in the overall/high-risk population.	High rate of ONJ (5.4% in D-CARE), indicating severe over-suppression.	“Frozen Bone” Failure: Aggressive blockade induces a “frozen” niche. The high toxicity suggests the disruption of physiologic coupling, creating a rigid, immunologically inert environment.	Gregoric et al.,^82^ Eisen et al.,^92^ Shapiro et al.^93^
ABCSG-18	niche-preserving modulation: less frequent dosing (e.g., Q6M) aligning with osteoporosis management.	significant improvement in DFS.	0% ONJ rate, indicating preservation of physiological remodeling.	normalization Success: The regimen mitigates bone loss without abrogating remodeling. The absence of ONJ suggests that the endosteal niche remains functional, maintaining dormancy signals.	Coleman et al.,^94^ Coleman et al.,^95^^,^^96^
EBCTCG Meta-analysis	context-dependent intervention: analysis stratified by menopausal status.	benefit strictly limited to postmenopausal women.	Efficacy coincides with the onset of estrogen-deprivation bone loss.	targeting the “Adipogenic Shift”: Suggests that efficacy is context-dependent. Drugs work by countering the specific metabolic defects of the “soil” (adipogenic shift), rather than killing the “seed.”	Barsky et al.^81^

Abbreviations: Q4W, every 4 weeks; Q6M, every 6 months; ONJ, osteonecrosis of the jaw; DFS, disease-free survival.

### Sarcopenia as a surrogate for niche degradation

While current pharmacotherapies effectively increase BMD, they do not address the systemic metabolic aging characterized by “anabolic resistance” in skeletal muscle.^85^^,^^101^ Sarcopenia represents more than simple functional decline; it constitutes a critical loss of mechanical input to the skeleton. The reduction in muscle mass lowers mechanical forces applied to the cortical surface, leading to the attenuation of osteogenic mechanotransduction. Crucially, this loss of mechanical stimulus does not merely suppress bone formation; it mechanically biases bone marrow MSCs toward an adipogenic lineage.^102^ This expansion of BMA creates a lipid-rich sanctuary for DTCs. Recent mechanistic investigations have suggested that bone marrow adipocytes exhibit robust metabolic activity, providing fatty-acid substrates that support breast cancer cell proliferation, migration, and mitochondrial metabolism.^19^ Such lipid provision has been further linked to enhanced therapeutic resistance and stemness regeneration in metastatic seeds.^103^

The premise that sarcopenia and adiposity actively alter tumor-bone metabolism is not merely a biological hypothesis; it is supported by large-scale clinical biomarker cohorts. Prospective imaging studies utilizing computed tomography have shown that severe skeletal muscle depletion, when combined with high adiposity, is associated with accelerated metastatic relapse and higher mortality in patients with nonmetastatic breast cancer.^104^ Crucially, this specific osteosarcopenic phenotype translates directly into systemic metabolic dysregulation. Randomized clinical trial data from breast cancer survivors confirm that sarcopenic obesity correlates directly with profound alterations in circulating metabolic biomarkers, including elevated systemic leptin, insulin, and chronic inflammatory cytokines.^105^ Therefore, sarcopenia may function not simply as a theoretical surrogate marker, but as a clinically relevant indicator and potential contributor to metabolic niche degradation that may facilitate tumor progression. This systemic loss of physiological reserve correlates with heightened chemotherapy toxicity, creating a feedback loop that further degrades the marrow niche.^106^^,^^107^ This implies that muscle atrophy and tumor metastasis will form a vicious cycle under the influence of systemic metabolic disorders, promoting the deterioration of the bone microenvironment while compromising treatment tolerance. Given the association with osteoporosis, muscle wasting may serve as a clinical indicator of underlying bone microenvironmental deterioration.

**Figure 2:**
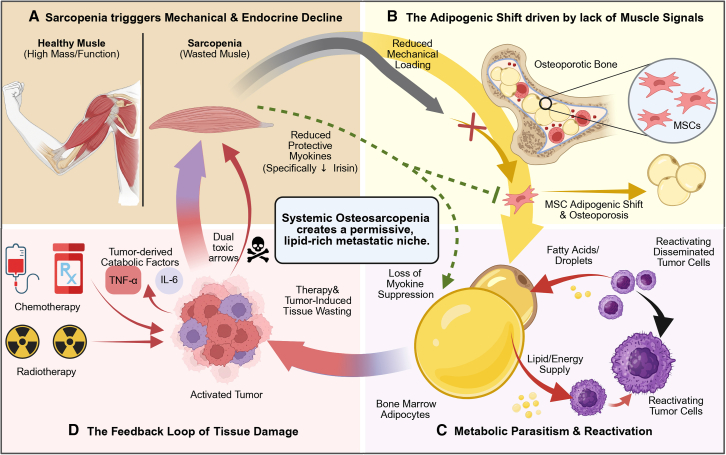
The osteosarcopenic vicious cycle: sarcopenia drives niche adipogenesis and tumor reactivation via mechanical and endocrine collapses Created in https://BioRender.com. The diagram illustrates how sarcopenia precipitates a metabolic collapse in the bone marrow, creating a permissive niche for breast cancer metastasis. (A) Sarcopenia, characterized by reduced muscle mass and function, leads to a dual deficit: diminished mechanical loading on the skeleton and a reduction in protective myokines, specifically Irisin. (B) The lack of mechanical stimulation biases the differentiation of bone marrow MSCs. The osteogenic lineage is suppressed, while the adipogenic lineage is unrestrained, leading to the expansion of BMAs and osteoporosis. (C) The expanded adipogenic niche acts as a “metabolic sanctuary”. Adipocytes fuel DTCs via lipid transfer to support mitochondrial β-oxidation. Concurrently, the systemic loss of Irisin removes a key suppressive signal, further promoting the transition of DTCs from dormancy to an active, proliferative state. (D) The reactivated tumor releases catabolic inflammatory factors. Furthermore, systemic treatments such as chemotherapy and radiotherapy induce off-target toxicity in musculoskeletal tissues. These factors exacerbate muscle wasting, closing the vicious cycle and perpetuating the deterioration of the metastatic niche.

Adiposity further exacerbates this condition. Clinical studies identify “sarcopenic obesity”-the combination of muscle wasting and fat accumulation-as a predictor of poor prognosis.^86^^,^^108^^,^^109^ This clinical phenotype aligns with the cellular mechanisms described previously, reflecting both the metabolic activity of marrow adipocytes^61^ and impaired osteocytic mechanotransduction.^79^^,^^80^ The complex interactions among endocrine deficiency, sarcopenia, and reduced mechanical loading inevitably lead to profound changes in the bone marrow environment. These changes collectively create a microenvironment that actively supports cancer progression. Consequently, using only BMD-targeted drugs may be less effective in preventing metastasis if systemic metabolic issues, such as osteosarcopenia, are not corrected. Long-term management of patients with breast cancer must address both local bone health and systemic physiological stability. This holistic approach can improve adjuvant bone-targeted therapy efficacy by targeting overall metabolic balance rather than bone density alone. This profound systemic interplay is conceptualized in Figure 2, which outlines the “osteosarcopenic vicious cycle”-illustrating how sarcopenia-driven mechanical and endocrine collapses actively precipitate bone marrow adipogenesis and subsequent tumor reactivation.

### Bridging the translational gap via metabolic co-targeting

**Figure 3:**
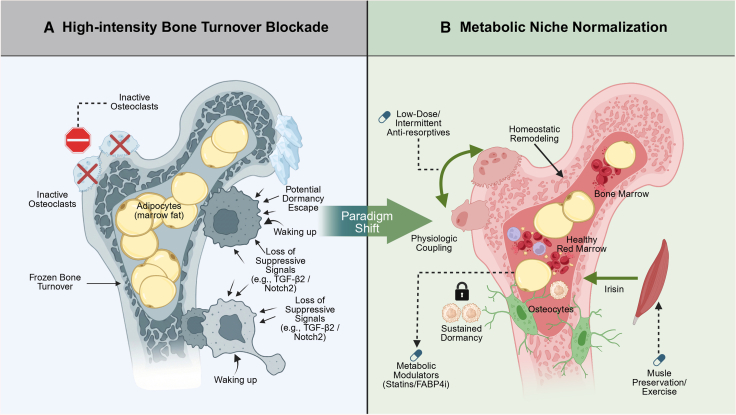
Conceptual framework illustrates the paradigm shift from a bone hardening strategy to niche normalization Created in https://BioRender.com. (A) High-intensity anti-resorptive blockade results in abrogated bone turnover and complete osteoclast inactivation. This microenvironmental state is characterized by the accumulation of marrow adiposity and the concurrent loss of osteoblast- and stromal-derived dormancy-maintaining signals, including Notch2-associated endosteal interactions and TGFβ2-dependent stromal cues, thereby facilitating the reactivation of disseminated tumor cells. (B) A niche-normalizing approach aims to restore homeostatic remodeling rather than inhibiting turnover completely. This strategy involves physiologic dosing of anti-resorptives to maintain osteoclast-osteoblast coupling integrated with metabolic interventions, including Irisin-mediated muscle preservation and adipocyte targeting. Restoration of a healthy bone marrow microenvironment and functional osteocytic connectivity promotes the sustained quiescence of tumor cells.

The clinical benefit of bone-targeted therapy depends on long-term patient adherence. Low-dose intermittent dosing regimens may not only improve patient adherence but also avoid the “frozen bone” state, thereby sustaining the suppressive capacity of the bone niche. When selecting a bone protection strategy, clinicians should balance efficacy against safety to ensure treatment continuity. Boosting treatment tolerability helps maintain therapy and improves long-term outcomes in patients with breast cancer.^110^ Beyond dosing schedules, future strategies may involve synergistic combinations that address metabolic factors. Lipophilic statins may synergize with RANKL inhibition, potentially by simultaneously targeting the metabolic machinery of both the osteoclast and the dormant tumor cell through the mevalonate pathway.^111^ Bridging this translational gap requires a fundamental evolution of clinical trial architectures beyond monolithic bone hardening protocols. Establishing specific methodological shifts will ensure that future trial designs accurately account for the systemic metabolic status of each patient. Implementing comprehensive patient stratification represents the first critical step, moving beyond standard bone mineral density scans. Future clinical trials should mandate baseline body composition imaging using routine computed tomography scans to calculate the skeletal muscle index alongside magnetic resonance imaging to quantify bone marrow adiposity. Integrating routine body-composition assessment may help identify high-risk osteosarcopenic phenotypes before randomization, ensuring that metabolic vulnerability serves as a primary stratification factor.^112^^,^^113^^,^^114^ Expanding primary endpoints to include multidimensional biomarkers constitutes the second essential shift. Beyond traditional bone turnover markers such as the cross-linked C-telopeptide of type I collagen and procollagen type 1 N-terminal propeptide, trials must prospectively monitor systemic metabolic signatures. Tracking the dynamic changes of adipokine ratios, specifically the balance between leptin and adiponectin, and circulating senescence-associated secretory phenotype factors, including interleukin 6, will more accurately reflect the metabolic permissiveness of the bone marrow niche over time.^113^ Adopting adaptive clinical trial designs allows for the evaluation of targeted metabolic interventions alongside bone-modifying agents. Future protocols should test the synergistic efficacy of combining physiologic dose antiresorptive medications with metabolic modulators, such as lipophilic statins or fatty acid binding protein 4 inhibitors. Additionally, incorporating structured resistance exercise interventions specifically tailored for metabolically compromised subgroups provides a holistic approach to restoring microenvironmental homeostasis. Integrating these precise systemic metabolic parameters into trial designs will facilitate the translation of preclinical mechanistic discoveries into durable clinical prevention.^105^ This translational roadmap necessitates a fundamental conceptual leap, as illustrated in Figure 3, which explicitly contrasts the limitations of traditional high-intensity “bone-hardening” strategies with the comprehensive, metabolically restorative approach of “niche normalization.”

## Conclusion and future perspectives

The clinical challenge of late-onset bone metastasis forces us to reconsider the “seed and soil” hypothesis through a metabolic lens. As we have argued, skeletal aging is not a passive decay but an active transformation into a pro-inflammatory, lipid-rich sanctuary that shelters dormant tumor cells. The failure of high-dose anti-resorptive therapies to prevent metastasis highlights a critical blind spot: strictly increasing bone mass does not necessarily normalize the marrow’s metabolic dysfunction. We might need to move beyond “bone hardening” agents to therapies that restore microenvironmental homeostasis—potentially by repurposing metabolic modulators like statins or FABP inhibitors, but clinical trials are still needed to verify their efficacy and safety. By treating the osteosarcopenic niche rather than just the tumor, we may finally be able to extend the dormancy of disseminated cells indefinitely.

## Ethics approval and consent to participate

This present study received approval from the institutional review board of the Affiliated Hospital of Zunyi Medical University, and all methods were performed in accordance with the relevant guidelines and regulations. Since this is a review, patient participation and informed consent are not applicable.

## Acknowledgments

This research was supported by the Guizhou Provincial Basic Research Program (Qiankehe-ZK[2024]-329) and the Guizhou Provincial Health Commission Science and Technology Fund (gzwkj2025-480).

## Author contributions

Conceptualization, D.H. and Q.J.; data curation, D.H. and Q.J.; formal analysis, D.H.; funding acquisition, H.X.; investigation, H.X.; methodology, D.H.; project administration, D.H. and Q.J.; supervision, H.X.; visualization, H.X.; writing – original draft, D.H. and Q.J.; writing-review and editing, H.X.

## Declaration of interests

The authors declare that there is no conflict of interest.
